# Cardiovascular outcomes in Parkinson’s disease patients from a retrospective cohort study

**DOI:** 10.1038/s41598-024-72549-y

**Published:** 2024-09-20

**Authors:** Subin Lim, Yun Jin Yum, Jong-Ho Kim, Chan-Nyoung Lee, Hyung Joon Joo, Do-Young Kwon

**Affiliations:** 1 Division of Cardiology, Department of Internal Medicine, Korea University Anam Hospital, Seoul, Republic of Korea; 2 Department of Biostatistics, Korea University College of Medicine, Seoul, Republic of Korea; 3 Korea University Research Institute for Medical Bigdata Science, Korea University College of Medicine, Seongbuk-Gu, Seoul, Republic of Korea; 4 Department of Neurology, Korea University Anam Hospital, Seoul, Republic of Korea; 5 Department of Medical Informatics, Korea University College of Medicine, Seoul, Korea; 6 Department of Neurology, Korea University Ansan Hospital, 123 Jeokgeum‐ro, Danwon‐gu, Ansan‐si, Gyeonggi‐do Republic of Korea

**Keywords:** Parkinson’s disease, Cardiovascular diseases, Risk factors, Prognosis, Propensity score, Parkinson's disease, Cardiology

## Abstract

Parkinson’s disease (PD) reports high rates of morbidity and mortality, but the risk of adverse cardiovascular outcomes in patients with PD has not been fully elucidated. This bi-center retrospective cohort study using the electronic health records (EHR) database of two tertiary hospitals screened a total of 327,292 subjects who visited the outpatient clinic, and 1194 patients with PD were propensity score-matched with a control population. The primary outcome was the occurrence of major adverse cardiovascular events (MACE). Key secondary outcomes included all-cause death, cardiovascular (CV) death, stroke, myocardial infarction (MI), heart failure hospitalization and 30-day CV death. After PS matching, MACE occurrence was not significantly different between PD and non-PD groups (18.2% vs. 17.5%, log-rank p = 0.98). Key secondary outcomes were also similar between the two groups. In patients with PD, MACE rate, and also CV risk score, were higher in patients with more severe PD (according to Hoehn and Yahr scale and unified Parkinson’s disease rating scale), and after multivariable analysis, PD severity was not an independent predictor of MACE. Patients with PD are at an increased risk of adverse cardiovascular outcomes, but the contribution from other common CV risk factors cannot be ignored. The management of prevalent CV risk factors is therefore important in mitigating adverse outcomes among patients with PD.

## Introduction

Parkinson’s disease (PD) is the second most common neurodegenerative disease after Alzheimer’s disease, with approximately 6 million or more patients with PD worldwide in 2016^1,2^. It has also been estimated that by 2030, the number of PD patients will increase to 8–10 million^3^. The prevalence of PD is increasing partly due to an aging population, an increase in the duration of the disease, and changes in socio-environmental risk factors^4^. In addition, given that PD is essentially a disease of the elderly, many patients with PD have chronic diseases such as high blood pressure and diabetes, pertaining to a high cardiovascular risk.

People with PD are estimated to survive on average 7 to 14 years after diagnosis, and while the mortality rate in patients with PD increases with age, it is more than double that of the general population^5,6^. However, the contributors to the increased mortality are not as sharply defined. In a 13.5-year follow-up study, although patients with PD had higher mortality rates compared with the general population, cerebrovascular and cardiovascular diseases did not appear to generate added risks^7^. On the other hand, another study reported that the risk of death from cardiovascular and cerebrovascular causes was greater in patients with PD than in the general population^8^. A recent meta-analysis of 9 cohort studies and 2 case–control studies suggested that PD increased the risk of stroke, with no difference in myocardial infarction (MI) and cardiovascular mortality^9^. Data is still limited, and further elaboration is needed.

Accurate data and robust statistical considerations would be important to unambiguously analyze the risk of cardiovascular hard endpoints, such as cardiovascular death, for people with PD. This study aimed to compare and analyze hard endpoints of death, MI, and stroke as well as other clinical outcomes of common infections and cancer in patients with PD using precise electronic health record (EHR) data of tertiary hospitals.

## Methods

### Study design

This is a bi-center retrospective pooled cohort study using the EHR database of two tertiary hospitals in Korea. The dataset of the present study was extracted through direct querying using SQL (structured query language). The study was approved by the Institutional Review Board (IRB) of each hospital (the IRB of Korea University Anam Hospital and the IRB of Korea University Ansan Hospital). Written informed consent was waived by the IRB of each hospital (the IRB of Korea University Anam Hospital and the IRB of Korea University Ansan Hospital), due to the use of a retrospective study design of anonymized data with minimal risk to study subjects. The study also complied with the Declaration of Helsinki. The study designs and results are described according to the STROBE (Strengthening the Reporting of Observational studies in Epidemiology) guidelines.

### Study population

**Fig. 1:**
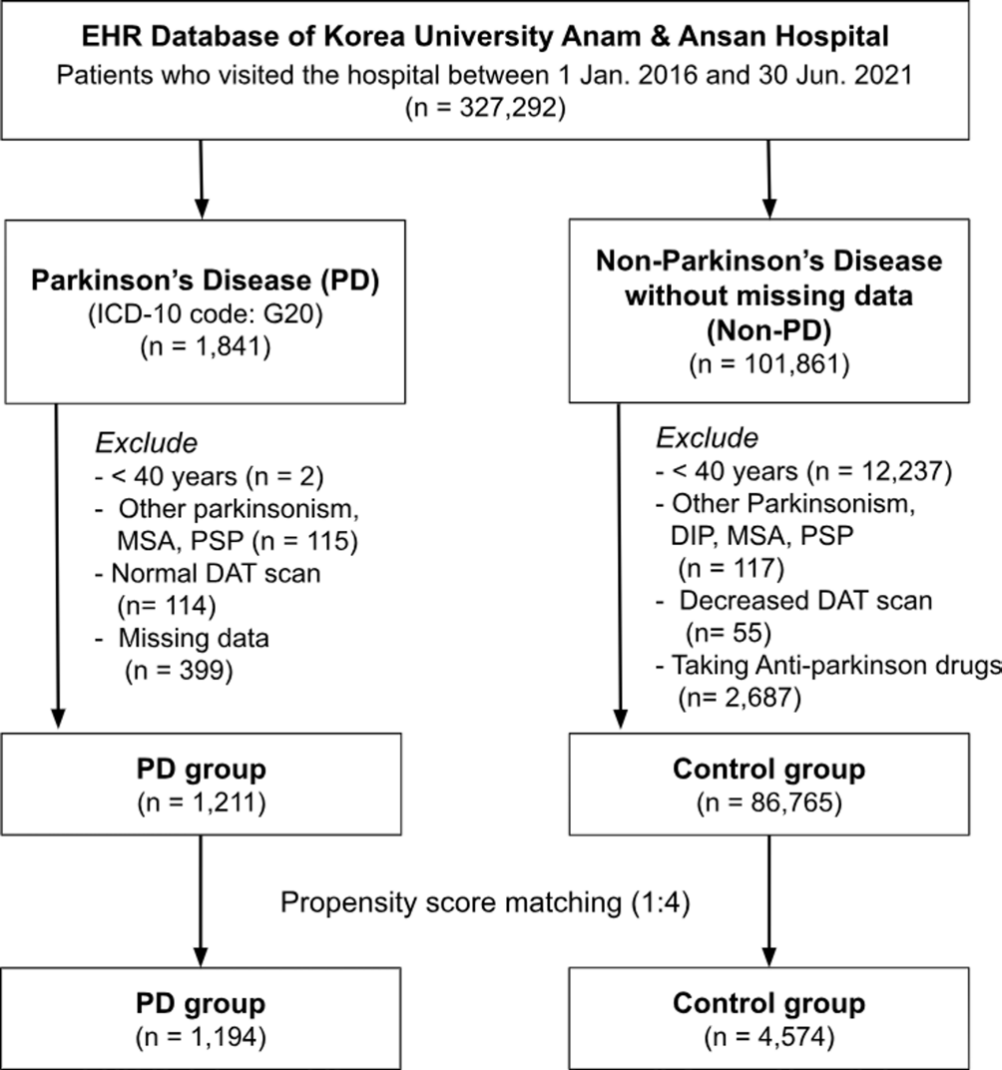
Study flowchart. From 327,292 patients with hospital visits between January 2016 and June 2021, a final propensity score-matched cohort of 5768 patients was selected for analysis between PD and non-PD patients. *EHR* electronic health records, *DAT* dopamine transporter, *MSA* multiple system atrophy, *PD* Parkinson’s disease, *PSP* progressive supranuclear palsy.

For enrolment, 327,292 patients who made a visit to an outpatient clinic in either Korea University Anam or Ansan Hospital between January 1, 2016, and June 30, 2021, were screened from the EHR database (Fig. 1). The PD group included patients with (1) at least 4 visits with ICD-10 diagnostic code for PD (G20) and received PD medications for more than 90 days; or (2) one or more visits with an ICD-10 diagnostic code for PD (G20) over the same period and a 18F-N-(3-fluoropropyl)-2β-carbon ethoxy-3β-(4-iodophenyl) nortropane positron emission tomography (18F-FP-CIT PET) scan showing decreased dopamine transporter (DAT) at posterior putamen. The index date was the first PD diagnosis date, and only the patients whose PD diagnosis was first made in the outpatient clinic were included. The exclusion criteria for the PD group were (1) ICD-10 diagnostic codes for other Parkinsonism (G21.1), multiple system atrophy (G23.2, G23.3, G90.3) or progressive supranuclear ophthalmoplegia (G23.1); (2) normal DAT finding at posterior putamen in the 18F-FP-CIT PET scan; (3) younger than 40 years, or (4) patients with missing values in the baseline clinical variables. Patients with prior admissions with an ICD-10 code for PD before the first outpatient clinic visit with PD diagnosis were excluded.

The control group comprised of patients without a history of diagnosis of any type of Parkinson’s disease over the same period as the PD group. The other exclusion criteria for the control group were (1) decreased DAT finding in 18F-FP-CIT PET scan; (2) prescription of Parkinson’s disease medications; (3) younger than 40 years; or (4) missing values in the baseline clinical variables. The index date was the first date for which cardiovascular risk assessment information including anthropometric information, blood pressure, blood glucose, lipid profile, hsCRP, and baseline laboratory results were all available.

Study groups were matched through propensity score matching, and finally, cardiovascular event rates were compared for 1194 patients in the PD group and 4574 patients in the control group (Supplemental Table S1). A minimum follow-up period of one year since the index date was required for all patients, and the patients were followed up for a median of 3.16 years (inter-quartile range, 1.52–4.63 years).

### Definitions and study endpoints

The primary outcome of the study was the occurrence of major adverse cardiovascular event (MACE). MACE was defined as a composite of cardiovascular death, stroke, MI, and hospitalization for heart failure. Key secondary endpoints included the individual components of MACE. Other secondary endpoints were the occurrence of non-cardiovascular outcomes, which included infectious diseases (pneumonia and urinary tract infection [UTI]) and malignant neoplasms. Hospitalization for heart failure was defined as NT-proBNP ≥ 300 pg/mL (≥ 600 pg/mL for atrial fibrillation) treated with furosemide at the time of admission^10^. MI was defined as chest pain or dyspnea and an increase in creatine kinase MB (CK-MB) level (> 99th percentile of upper reference limit) at least 2 times. Stroke was defined by the diagnosis code of cerebral infarction and confirmation of acute, subacute, or recent cerebral infarction on brain magnetic resonance imaging. Cardiovascular death was defined as cases with hospitalization due to heart failure, MI, or stroke within 30 days of death.

Hypertension was defined as systolic blood pressure ≥ 140 mmHg, diastolic blood pressure ≥ 90 mmHg, anti-hypertensive medications within 30 days of the index date or having a diagnosis code for hypertension. Diabetes was defined as taking oral hypoglycemic drugs or insulin, HbA1c ≥ 6.5%, blood glucose ≥ 126 mg/dL, or having a diabetes diagnosis code. Dyslipidemia was defined as taking statins or ezetimibe, serum total cholesterol ≥ 250 mg/dL, low-density lipoprotein-cholesterol (LDL-C) ≥ 160 mg/dL, triglyceride ≥ 200 mg/dL, high-density lipoprotein-cholesterol (HDL-C) < 40 mg/dL or a diagnosis code for dyslipidemia. Chronic kidney disease (CKD) was defined as an estimated glomerular filtration rate (eGFR) < 60 mL/min/1.73 m^2^ calculated using serum creatinine level or proteinuria ≥ 1 + on the routine urine analysis.

Cardiovascular risk was assessed based on the risk categories suggested by the European Society of Cardiology (ESC) guidelines on cardiovascular disease prevention and the Systemic Coronary Risk Evaluation (SCORE2) model, upon which patients were classified into low to moderate, high, and very high-risk groups (Supplemental Table S2)^11,12^.

Clinical outcomes including cardiovascular outcomes, malignant neoplasm and infections were classified using ICD-10 codes (Supplemental Table S3). For assessment of PD severity, the Hoehn and Yahr (H&Y) scale and Unified Parkinson’s Disease Rating Scale-III (UDPRS-III) were used. The scores of each patient were classified into lowest, middle, and highest tertiles. The UDPRS-III and H&Y scales were verified by two independent researchers.

### Statistical analysis

Baseline characteristics are shown as the mean ± standard deviation or number (%). Chi-square test and Student’s t-test were used to compare the categorical variables and continuous variables between the groups. To reduce the effect of selection bias, we conducted a propensity score (PS) matching analysis to compare the PD and non-PD groups. For the PS matching, the likelihood of being diagnosed with PD was assessed using multivariable logistic regression. The patients were matched at a 1:4 ratio using the greedy nearest neighbor matching, with a caliper width equal to 0.2 of the standard deviation of the logit PS. Baseline characteristics described in Table 1, use of common medications (anti-hypertensive drugs, antidiabetic drugs, statins and diuretics) lipid profile and future cardiovascular risk score as calculated by the SCORE2 model were included in the model for PS matching analysis. The balance of baseline features between the PD group and the control group was assessed; a standardized mean difference of < 0.1 indicated a negligible difference.

**Table 1:** Baseline characteristics of patients according to PD status.

	PD (n = 1211)	Non-PD (n = 86,715)	*P*-value
Age (years)	73.1 ± 10.0	62.8 ± 11.7	< 0.01
Male (n, %)	530 (43.8)	44,467 (51.3)	< 0.01
Alcohol (n, %)	218 (18.0)	14,988 (17.3)	0.51
Smoking (n, %)	129 (10.7)	11,471 (13.2)	< 0.01
Hypertension (n, %)	783 (64.7)	55,502 (64.0)	0.64
Diabetes (n, %)	524 (43.3)	33,132 (38.2)	< 0.01
Dyslipidemia (n, %)	770 (63.6)	60,036 (69.2)	< 0.01
CKD (n, %)	298 (24.6)	21,113 (24.4)	0.83
Atrial fibrillation (n, %)	76 (6.3)	6480 (7.5)	0.12
Prior myocardial infarction (n, %)	40 (3.3)	4452 (5.1)	< 0.01
Prior heart failure (n, %)	10 (0.8)	3323 (3.8)	< 0.01
Prior stroke (n, %)	238 (19.7)	8175 (9.4)	< 0.01
Prior PCI (n, %)	30 (2.5)	7645 (8.8)	< 0.01
Systolic blood pressure (mmHg)	127.8 ± 17.5	129.0 ± 16.3	0.02
Diastolic blood pressure (mmHg)	74.9 ± 11.3	77.1 ± 12.0	< 0.01
Heart rate (bpm)	79.6 ± 13.3	80.9 ± 17.0	0.03
Body mass index (kg/m^2^)	25.0 ± 18.8	25.8 ± 19.6	0.21
Hoehn–Yahr stage	2.3 ± 1.1	–	–
UPDRS scale III	25.9 ± 15.8	–	–
Parkinson’s disease drugs			
Levodopa (± DDCI and/or COMT inhibitors) (n, %)	1081 (89.3)	–	–
Dopamine agonist (n, %)	548 (45.3)	–	–
MAO-B inhibitor (n, %)	494 (40.8)	–	–
Amantadine (n, %)	225 (18.6)	–	–
Anticholinergics (n, %)	336 (27.8)	–	–
Anti-hypertensive medication			
RAS inhibitor (n, %)	494 (40.8)	35,920 (41.4)	0.66
DHP-CCB (n, %)	435 (35.9)	28,528 (32.9)	0.03
Beta-blocker (n, %)	455 (37.6)	24,090 (27.8)	< 0.001
Diuretics (n, %)	304 (25.1)	22,640 (26.1)	0.43
Anti-lipidemic medication			
Statin (n, %)	532 (43.9)	43,333 (50.0)	< 0.01
Anti-diabetic medication			
OHA (n, %)	255 (21.1)	22,476 (25.9)	< 0.01
Insulin (n, %)	128 (10.6)	10,587 (12.2)	0.08
Laboratory finding			
Potassium (mEq/L)	4.2 ± 0.4	4.3 ± 0.4	< 0.01
Hemoglobin (g/dL)	13.1 ± 1.6	13.7 ± 1.7	< 0.01
Creatinine (mg/dL)	0.9 ± 0.3	1.0 ± 0.8	0.06
eGFR (mL/min/1.73 m^2^)	80.3 ± 22.7	85.3 ± 23.0	< 0.01
Total cholesterol (mg/dL)	166.9 ± 38.3	170.8 ± 42.0	< 0.01
HDL-cholesterol (mg/dL)	49.0 ± 12.9	51.1 ± 13.6	< 0.01
LDL-cholesterol (mg/dL)	102.7 ± 32.5	104.4 ± 36.0	0.11
Triglyceride (mg/dL)	120.2 ± 64.3	141.1 ± 99.1	< 0.01
hsCRP (mg/L)	1.0 ± 1.8	1.1 ± 1.9	0.12
Glucose (mg/dL)	122.8 ± 49.3	115.2 ± 35.6	< 0.01
HbA1c (%)	6.2 ± 1.1	6.4 ± 1.3	< 0.01
CV risk group* (n, %)			< 0.01
Low-moderate risk	187 (15.4)	26,079 (30.1)	
High risk	442 (36.5)	29,725 (34.3)	
Very high risk	582 (48.1)	30,911 (35.7)	

Values are presented as mean ± standard deviation or number (%).

*CKD* chronic kidney disease, *COMT* Catechol-O-methyltransferase, *CV* cardiovascular, *DDCI* dopa decarboxylase inhibitor, *DHP-CCB* dihydropyridine calcium channel blocker, *eGFR* estimated glomerular filtration rate, *HbA1c* haemoglobin A1c, *HDL* high-density lipoprotein, *hsCRP* high-sensitivity C-reactive protein, *LDL* low-density lipoprotein, *MAO-B* monoamine oxidase B, *PCI* percutaneous coronary intervention, *PD* Parkinson’s disease, *RAS* renin-angiotensin system, OHA, oral hypoglycemic agents, SCORE2, systematic coronary risk assessment 2, *SMD* standardised mean difference, *UPDRS* Unified Parkinson’s Disease Rating Scale.

*Cardiovascular 10-year mortality risk.

The probabilities for clinical outcomes were calculated by the Kaplan–Meier estimates and compared by log-rank test. Multivariable Cox regression analysis was performed adjusting for clinical variables of age, sex, smoking status, alcohol consumption status, hypertension, diabetes mellitus, dyslipidemia, chronic kidney disease, atrial fibrillation, prior MI, prior stroke, prior heart failure (HF), and the use of common medications (statin, diuretics, and antihypertensive medications). Among patients with PD, an additional analysis of the occurrence of clinical outcomes according to PD severity was performed. Hazard ratio for clinical outcomes in PD patients was obtained by Cox regression analysis. Cox regression analysis was also used for analysis of the non-cardiovascular outcomes of infections and malignant neoplasms. All analyses were performed using SAS 9.4 (SAS Institute Inc., Cary, NC, USA) program.

## RESULTS

### Baseline characteristics

A total of 5768 patients were selected for analysis in our study. (Table 1) At baseline, patients with PD were older, with a higher prevalence of females and fewer smokers. The PD group were also more diabetic and less likely to have dyslipidemia, prior MI, prior HF, or prior PCI history. Prior stroke history was more prevalent in patients with PD. Patients with PD had a mean Hoehn-Yahr stage of 2.3 ± 1.1 and a mean Unified Parkinson’s Disease Rating Scale (UDPRS-III) of 25.9 ± 15.8.

Total cholesterol and triglyceride levels were higher in the non-PD group. Risk of future cardiovascular diseases and mortality, as assessed by the SCORE2 prediction model, was higher in the PD group, with 58.9% of the PD group and 28.6% of non-PD population at very high risk, respectively.

### Cardiovascular outcomes in patients with PD

**Fig. 2:**
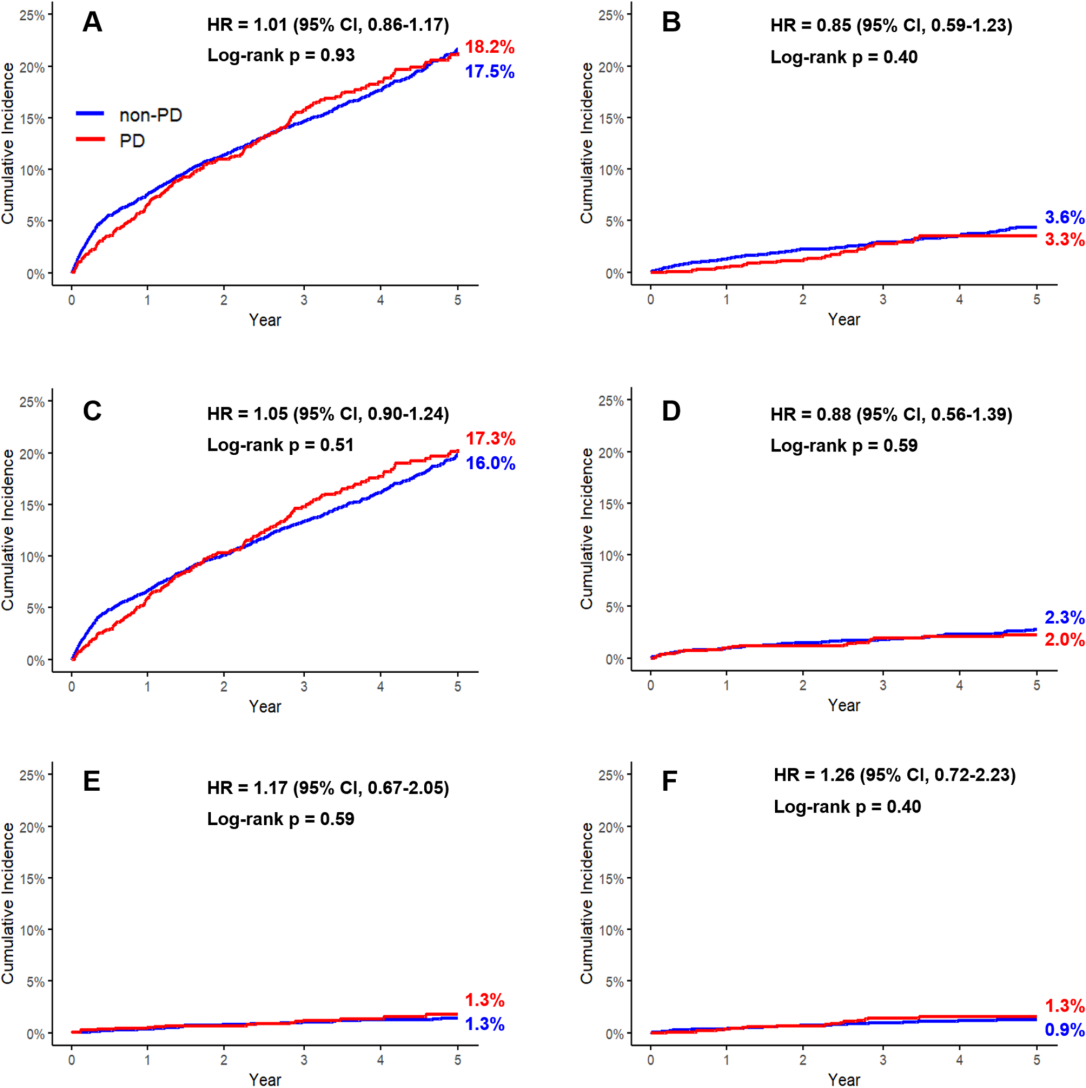
Clinical outcomes for PD versus non-PD patients. The Kaplan–Meier curves represent the cumulative incidence of each clinical outcome in PD and non-PD groups after PS matching. (**A**) MACE, (**B**) all-cause death, (**C**) HF, (**D**) MI, and (**F**) CV death. *CV* cardiovascular, *HF* heart failure, *MACE* major adverse cardiovascular events, *PD* Parkinson’s disease.

Before PS matching, MACE occurred in 17.1% of the patients with PD and 12.8% of the non-PD population (log-rank p < 0.01) (Supplemental Table S4). After PS matching, however, MACE occurred in 18.2% of patients with PD and 17.5% of the non-PD population (log-rank p = 0.98, Table 2, Fig. 2). Differences in all-cause death (3.3% vs. 3.6%, p = 0.39), CV death (1.3% vs. 0.9%, p = 0.31), stroke (1.3% vs. 1.3%, p = 0.64), MI (2.0% vs. 2.3%, p = 0.54), HF hospitalization (17.3% vs. 16.0, p = 0.63) and 30-day CV death (1.4% vs. 1.1%, p = 0.31) were also insignificant between the two groups after PS matching.

**Table 2:** Cardiovascular outcomes according to PD status after propensity score matching.

	PD (n = 1194)	Non-PD (n = 4574)	*P-value
MACE	217 (0.182; 0.16–0.20)	801 (0.175; 0.16–0.19)	0.98
CV death	15 (0.013; 0.01–0.02)	42 (0.009; 0.01–0.01)	0.31
Stroke	16 (0.013; 0.01–0.02)	53 (0.012; 0.01–0.02)	0.64
Myocardial infarction	24 (0.020; 0.01–0.03)	104 (0.023; 0.02–0.03)	0.54
Hospitalization for HF	206 (0.173; 0.15–0.19)	733 (0.16; 0.15–0.17)	0.63
All-cause death	39 (0.033; 0.02–0.04)	165 (0.036; 0.03–0.03)	0.39

Values are presented as numbers (the Kaplan–Meier estimate of cumulative incidence; 95% CI).

MACE is a composite of CV death, stroke, myocardial infarction and hospitalization for heart failure.

*CI* confidence interval, *CV* cardiovascular, *HF* heart failure, *HR* hazard ratio, *MACE* major adverse cardiovascular events, *PD* Parkinson’s disease.

*p-value for the log-rank test.

In multivariable analysis, PD diagnosis was not a significant predictor of MACE (Hazard ratio [HR], 0.98; 95% confidence interval [CI], 0.84–1.14; p-value 0.80). (Table 3) Independent predictors of MACE included age (HR, 1.03; 95% CI, 1.02–1.04; p < 0.001), smoking status (HR, 1.60; 95% CI, 1.31–1.95; p < 0.001), drinking status (HR, 1.66; 95% CI, 1.39–1.98; p < 0.001), DM (HR, 1.30; 95% CI, 1.14–1.47; p < 0.001), CKD (HR, 1.47; 95% CI, 1.28–1.68; p < 0.001), AF (HR, 2.28; 95% CI, 1.90–2.75; p < 0.001), prior HF (HR, 1.46; 95% CI, 1.14–1.86; p = 0.002) and prior MI (HR, 6.44; 95% CI, 4.57–9.07; p < 0.001).

**Table 3:** Risk of MACE in the PS-matched cohort.

	Univariable		Multivariable	
	HR (95% CI)	P-value	HR (95% CI)	P-value
Age	1.05 [1.04;1.05]	< 0.001	1.03 [1.02;1.04]	< 0.001
Sex	0.83 [0.73;0.94]	0.00294	1.13 [0.98;1.31]	0.09819
Smoking status	2.19 [1.87;2.56]	< 0.001	1.60 [1.31;1.95]	< 0.001
Drinking status	2.04 [1.77;2.34	< 0.001	1.66 [1.39;1.98]	< 0.001
PD	1.01 [0.86;1.17]	0.93	0.98 [0.84;1.14]	0.79857
Hypertension	1.80 [1.55;2.09]	< 0.001	1.06 [0.87;1.30]	0.54141
DM	1.56 [1.38;1.77]	< 0.001	1.30 [1.14;1.47]	< 0.001
CKD	2.05 [1.80;2.33]	< 0.001	1.47 [1.28;1.68]	< 0.001
Dyslipidemia	1.45 [1.26;1.67]	< 0.001	1.29 [1.07;1.55]	0.00643
AF	3.39 [2.85;4.03]	< 0.001	2.28 [1.90;2.75]	< 0.001
Prior HF	2.79 [2.22;3.51]	< 0.001	1.46 [1.14;1.86]	0.00258
Prior MI	8.41 [6.05;11.69]	< 0.001	6.44 [4.57;9.07]	< 0.001
Prior stroke	1.40 [1.21;1.62]	< 0.001	0.95 [0.82;1.11]	0.53526
Statin	1.28 [1.13;1.46]	< 0.001	0.78 [0.66;0.93]	0.00502
Diuretics	2.50 [2.20;2.83]	< 0.001	1.57 [1.36;1.82]	< 0.001
RAS blocker	1.78 [1.57;2.02]	< 0.001	0.92 [0.77;1.10]	0.36069
DHP-CCB	1.64 [1.44;1.85]	< 0.001	1.13 [0.96;1.32]	0.14358
Beta-blocker	1.79 [1.58;2.03]	< 0.001	1.19 [1.04;1.37]	0.01287

Analysis by Cox proportional hazards model.

*AF* atrial fibrillation, *CI* confidence interval, *CKD* chronic kidney disease, *DHP-CCB* dihydropyridine calcium channel blockers, *DM* diabetes mellitus, *HF* heart failure, *HR* hazard ratio, *MACE* major adverse cardiovascular events, *MI* myocardial infarction, *PD* Parkinson’s disease, *PS* propensity score, *RAS* renin-angiotensin system.

### Disease severity and PD

In the PD group, with increasing disease severity, the occurrence of MACE also increased. As shown in Table 4, the occurrence of MACE ranged from 14.5% in the lowest tertile to 29.4% in the highest tertile according to H&Y scale (p_trend_ < 0.01). Similar tendency was observed when patients were classified by UPDRS-III, with MACE occurring in 12.5% of the lowest tertile group and reaching up to 23.3% in the highest tertile (p_trend_ = 0.07).

**Table 4:** MACE occurrence and the co-presence of CV risk factors, according to PD severity**.**

H&Y scale				
	Lowest tertile (N = 166, 17.1%)	Middle tertile (N = 540, 55.6%)	Highest tertile (N = 265, 27.3%)	p for trend
MACE	21 (12.7)	71 (13.2)	73 (27.6)	< 0.01
CV risk				
Low-moderate	48 (28.9)	100 (18.5)	17 (6.4)83 (31.3)	< 0.01
High	67 (40.4)	219 (40.6)		
Very high	51 (30.7)	221 (40.9)	165 (62.3)	

UPDRS-III				
	Lowest tertile (N = 241, 24.7%)	Middle tertile (N = 474, 48.5%)	Highest tertile (N = 262, 26.8%)	p for trend
MACE	28 (11.6)	73 (15.4)	59 (22.5)	< 0.01
CV risk				
Low-moderate	64 (26.6)	75 (15.8)	22 (8.4)85 (32.4)	< 0.01
High	106 (44.0)	184 (38.8)	184 (38.8)	
Very high	71 (29.5)	215 (45.4)	155 (59.2)	

*CV* cardiovascular, *H&Y* Hoehn and Yahr scale, *MACE* major adverse cardiovascular events, *PD* Parkinson’s disease, *UPDRS-III*, Unified Parkinson’s Disease Rating Scale.

The CV risk factor score, however, was also higher in the more severe PD groups. The highest tertiles for PD severity scales coincided with the highest percentage of patients with very high CV risk. According to the H&Y scale, patients with very high CV risk comprised 62.3% of the highest tertile, while only 30.7% of the patients in the lowest tertile presented with very high CV risk. Similarly, with UPDRS-III, 59.2% of the patients in the highest tertile had very high CV risk, while only 29.5% of the lowest tertile patients showed very high CV risk. In a separate multivariable analysis amongst patients with PD, PD severity was not an independent predictor of MACE, using either the H&Y scale or the UPDRS-III score (Supplemental Table S5a and S5b).

### Non-cardiovascular outcomes in patients with PD

For the analysis of non-cardiovascular outcomes in PD and non-PD patients, we checked the occurrence of malignant neoplasms and common infectious disease (pneumonia and urinary tract infections (UTI)) in PD and control group patients. The occurrence of certain types of malignant neoplasms were higher in the non-PD population, namely lung, gastric, liver and colorectal cancers (Supplemental Table S6). The occurrences of prostate and breast cancers were not significantly different between the two groups. The occurrences of UTI (7.2% vs. 4.1%, p < 0.01) and pneumonia (5.0% vs. 3.8%, p = 0.10) were both higher in the PD group, with statistical significance for UTI occurrences. Notably, admissions due to pneumonia (2.7% vs. 1.6%, p = 0.01) and UTI (2.4% vs. 0.6%, p < 0.01) were both significantly higher in the PD group. The 30-day mortality from both pneumonia (0.6% vs. 0.4%, p = 0.38) and UTI (0.2% vs. 0.04%, p = 0.15) were higher in people with PD, although without statistical significance. The 30-day mortality rate from either infectious disease was very low (pneumonia, 0.6% vs. 0.4%; UTI, 0.2% vs. 0.04%), with 25 deaths from pneumonia and 4 from UTI.

In Cox proportional hazards analysis, none of the CV outcomes were significantly increased for patients with PD (Supplemental Table S7). There was an increased risk of UTI in patients with PD (HR, 1.73; 95% CI, 1.33–2.24; p < 0.001). There was an overall decreased cancer risk in PD patients. Lung cancer (HR, 0.40; 95% CI, 0.19–0.83; p = 0.013), gastric cancer (HR, 0.39; 95% CI, 0.22–0.67; p < 0.001), colorectal cancer (HR, 0.26; 95% CI, 0.13–0.51; p < 0.001) and liver cancer (HR, 0.25; 95% CI, 0.13–0.49; p < 0.001) were less commonly manifest in patients with PD. There were no significant differences for prostate (HR, 0.83; 95% CI, 0.51–1.34; p <  = 0.44) or breast (HR, 0.57; 95% CI, 0.31–1.04; p = 0.07) cancers.

## DISCUSSION

The present PS-matched analysis compared cardiovascular outcomes between PD and non-PD patients using robust statistical methods. Our main findings were that 1) after PS matching, cardiovascular outcomes were not significantly different for PD and control patients; 2) in patients with PD, both higher disease severity and the presence of cardiovascular risk factors were associated with an increase in cardiovascular event rate; and 3) in patients with PD, PD severity alone was not an independent predictor of adverse cardiovascular events after multivariable adjustment. To the best of our knowledge, our study is the largest study using EHR data on the relationship between PD and cardiovascular outcomes. Our study is also notable for its inclusion of laboratory data and consideration of disease severity of PD.

Parkinson’s disease entails many comorbidities, and increased mortality in people with PD has been documented in numerous studies^7,8,13–16^. This was also evident in our study, as the observed crude mortality rate and the occurrence of other cardiovascular adverse events were significantly higher in the PD group. The specific contributors to the increased mortality, however, are often less clear, and the evidence is mixed for cardiovascular outcomes in patients with PD. In a 13.5-year follow-up study, people with PD died more than the control patients, but neither heart disease (27.3% vs. 35.3%, p = 0.70) nor stroke (20.5% vs. 22.1%, p = 1.00) were significantly different as the cause of death^14^. On the other hand, a 38-year follow-up study in people with PD has shown increased mortality rates from cerebrovascular and cardiovascular causes, with SMRs of 1.84 and 1.58, respectively^8^. Population-based studies from Taiwan and South Korea have also reported increased cardiovascular risks for the PD population^15,16^. According to the Taiwanese National Health Insurance data, during a 3-year follow-up, PD patients were at an increased risk of AMI and CV deaths^15^. Also, based on the Korean National Institute of Health data, PD patients had higher risks of cardiovascular events such as MI, ischemic stroke, and congestive heart failure^16^.

While the exact nature of the relationship between PD and adverse cardiovascular events is unclear, chronic non-communicable medical conditions such as DM or hypertension act as common risk factors for stroke, MI, heart failure, and mortality. In our study, patients with PD also tended to be older and have a higher prevalence of DM, dyslipidemia and CKD. The PD group consisted of a higher percentage of female patients, which contrasts to data from many Western studies but is unsurprising in a population predominantly East Asian in ethnicity. For reasons not completely clear, many Asian studies have reported higher incidences of female patients with PD^17,18^. In 2019, Park et al. reported the annual incidence of PD in Korea to be around 22 to 28 cases per 100,000 individuals, of which 42.3% were male^18^.

The results from our study show that although overall mortality and the occurrence of adverse cardiovascular outcomes were higher in patients with PD, events occurred at a similar rate for PD and non-PD patients after PS matching for demographic variables such as DM and hypertension. In multivariable analyses among patients with PD, baseline demographics and co-morbidities such as age, CKD, previous HF history and previous MI history were independent predictors of MACE, while PD severity was not, for either the H&Y scale or the UPDRS-III score. Age is a well-known risk factor for PD, and disease severity tends to increase as patients get older. The results from our study imply that the stage of disease progression on its own appears to be less involved with adverse cardiovascular outcomes in patients with PD.

Notably, in comparison of the non-cardiovascular outcomes, the incidence of common infections tended be more frequent in patients with PD while there was an overall reduced risk of malignant neoplasms, which agrees with the current evidence. The heightened sensitivity to infections in patients with PD, especially those with advanced PD, is well recognized, primarily resulting from decreased mobility and ability to excrete sputum. Regarding malignancies, this seemingly “protective” effect of PD on cancer risk has been observed in many previous studies^19,20^, which is also seen in reverse, whereby cancer patients appear to be at lower risk of developing PD^21^. A meta-analysis showed that an overall 27% decreased risk of cancer was observed in patients diagnosed with PD^22^. The exact mechanism behind this is yet to be elucidated, but the extensively proliferative nature of cancer pathology and the degenerative changes and cell deaths that characterize PD may stand on different ends of a spectrum regarding cell cycles^23^.

In most case–control studies of PD, no significant relationships between DM and PD were discernible, and a case–control study by Miyake et al. even reported a reduced risk of PD with DM^24,25^. However, in a meta-analysis of nine studies of both prospective cohort and case–control designs, DM was a risk factor for PD in prospective cohort studies, while the relationship was not so certain in case–control studies^26^. The pooled risk of DM for PD was 1.37 in prospective cohort studies, and the association remained significant after exclusion of participants who had coronary artery disease or cerebrovascular disease. Three large prospective cohort studies from different populations have found that patients with DM were at an increased risk of developing PD^27–29^. The discrepancy between cohort studies and case–control studies could be the result of PD being a relatively rare disease, and that most population samples of people with PD are very heterogenous in nature. Case–control studies may be more prone to selection and recall biases. Diabetes is an inflammatory, metabolic condition that affects both large and small blood vessels, with possible common shared mechanisms for cardiovascular diseases and PD.

Similar tendencies were found for hypertension; while case–control studies reported little association, or even reduced risk of PD with hypertension, a meta-analysis of seven cohort studies found that a prior diagnosis of hypertension was strongly associated with an elevated incidence of PD (RR = 1.799, 95% CI 1.066–3.037), which remained significant after adjusting for potential vascular confounders^25^. Possible mechanisms linking hypertension to cerebrovascular diseases such as Alzheimer’s disease, and potentially PD, include alterations in resting cerebral blood flow, inflammatory responses in hypertensive patients, and brain atrophy and white matter injury resulting from chronic exposure to high blood pressure^30,31^.

Although the differences between the PD and non-PD groups were insignificant for cardiovascular outcomes after PS matching in our study, MACE did increase for patients with PD with increasing disease severity, as well as the cardiovascular risk prediction score. As shown in the multivariable analysis of patients with PD, age, but not PD severity, was a significant predictor of MACE. This suggests that the association of disease severity with adverse cardiovascular events is likely accounted for by disease duration, which is in turn related to age. Thus, while PD itself may not be a potent risk factor for MACE, after a diagnosis of PD is made, the progressive deconditioning likely elicits a vicious cycle and causes increasing predisposition to adverse outcomes. Additionally, considering the higher prevalence of cardiovascular risk factors and comorbidities in patients with PD, it is understandable that addressing these risk factors presumably improves the prognosis for patients with PD. Advancement of traditionally cardiovascular medicine such as glucagon-like peptide-1 (GLP-1) analogues in treatment of PD, for example, suggests that cardiovascular diseases and PD may share similar pathophysiologic aspects, enhancing the need to address CV risk factors in patients with PD^32,33^.

Specifically, the increase in adverse cardiovascular events may also be related to the lack of mobility associated with advanced PD, which are also prominent in older-age patients. Functional mobility impairment becomes more severe in patients with PD as the duration of the disease becomes more prolonged, often leading to bed-ridden statuses for a large proportion of time^34^. Being immobile brings about a range of unfavorable cardiovascular responses such as venous stasis and an increased risk of thromboembolism, decreased cardiac reserve, orthostatic hypotension, and cardiac deconditioning due to lack of exercise^35^. Some PD medications are also associated with potential cardiac effects such as QT-prolongation on electrocardiogram, tendency for ventricular arrhythmia or ischemic heart disease^36^. Increased dosing and the number of drugs in advanced PD patients may thus make advanced PD patients more susceptible to cardiac events or sudden death. Patients with other medical comorbidities are also likely to be on multiple medications, which may produce unintended side effects or interactions with medications for PD.

This study has some limitations. First, being a retrospective cohort study, the possibility of selection bias cannot be ignored. However, while the preponderance to the elderly and the frailty of the PD population may limit prospective or randomized studies regarding PD, the use of EHR data allows for the selection of a reasonable population size. Second, the patient group consisted solely of PD patients. Cautions are therefore needed when extrapolating the results to other forms of PD, such as multiple system atrophy or progressive supranulcear palsy. Third, the assessment of PD severity was based on the reported H&Y scale and UPDRS-III scores, which only show the total scores. The specific sub-categories of the severity scores were therefore not available for analysis. In future research, subgroup analyses of the sub-categories may provide further insight into which features of PD relate most to adverse cardiovascular outcomes.

Fourth, the study design allowed for the inclusion of patients with PD diagnosis made during a specific period of time. Therefore, those who were more recently diagnosed with PD, or those who have recently commenced treatment for PD were not included in the study. Fifth, most of the patients were of Korean ethnicity, limiting extrapolation of the results to other ethnic groups. Cardiovascular risk tends to be lower in East Asian populations, including Koreans. A report by the Center for Disease Control and Prevention has shown that Korean people living in the United States show lower prevalence of diabetes and coronary heart diseases compared with most other ethnicities^37^. The prevalence of PD also appears to be similar or lower in Asian populations, which is neutral to our results^38^. The lower prevalence of both cardiovascular diseases and PD in Korea may lead to an underestimation of cardiovascular risks in patients with PD. Global follow-up studies are therefore warranted to better elucidate the differential risks of developing cardiovascular diseases in patients with PD from different ethnic backgrounds.

## Conclusion

Patients with PD exhibit a heightened risk of morbidity and mortality compared with those without. However, it is important to acknowledge that other risk factors such as DM or HTN may contribute significantly towards the increased risk. Greater care should be taken to fully manage co-prevalent cardiovascular risk factors in patients with PD, for mitigation of adverse cardiovascular events.

## Supplementary Information

Supplementary Tables.

## Data Availability

The data used to support the findings of this study are available from the corresponding author upon reasonable request.

## Abbreviations

CV: Cardiovascular
H&Y: Hoehn and Yahr scale
MACE: Major adverse cardiovascular events
MI: Myocardial infarction
PD: Parkinson’s disease
PS: Propensity score
SCORE: Systemic Coronary Risk Evaluation model
UPDRS III: Unified Parkinson’s disease rating scale III
