# Publisher Correction: Cardiovascular outcomes in Parkinson’s disease patients from a retrospective cohort study

**DOI:** 10.1038/s41598-024-76160-z

**Published:** 2024-10-28

**Authors:** Subin Lim, Yun Jin Yum, Jong-Ho Kim, Chan-Nyoung Lee, Hyung Joon Joo, Do-Young Kwon

**Affiliations:** 1 Division of Cardiology, Department of Internal Medicine, Korea University Anam Hospital, Seoul, Republic of Korea; 2 Department of Biostatistics, Korea University College of Medicine, Seoul, Republic of Korea; 3 Korea University Research Institute for Medical Bigdata Science, Korea University College of Medicine, Seongbuk-Gu, Seoul, Republic of Korea; 4 Department of Neurology, Korea University Anam Hospital, Seoul, Republic of Korea; 5 Department of Medical Informatics, Korea University College of Medicine, Seoul, Korea; 6 Department of Neurology, Korea University Ansan Hospital, 123 Jeokgeum‐ro, Danwon‐gu, Ansan‐si, Gyeonggi‐do Republic of Korea

Correction to: *Scientific Reports*10.1038/s41598-024-72549-y, published online 20 September 2024

The original version of this Article contained an error in the grant number listed in the Funding section.

“This research was supported by the grants from the Medical data-driven hospital support project through the Korea Health Information Service (KHIS), funded by the Ministry of Health & Welfare, Republic of Korea and the MSIT (Ministry of Science and ICT), Korea, under the ICAN (ICT Challenge and Advanced Network of HRD) program (IITP-2023-RS-2022–00156439) supervised by the IITP (Institute of Information & Communications Technology Planning & Evaluation).”

now reads:

“This research was supported by the grants from the Medical data-driven hospital support project through the Korea Health Information Service (KHIS), funded by the Ministry of Health & Welfare, Republic of Korea and the MSIT (Ministry of Science and ICT), Korea, under the ICAN (ICT Challenge and Advanced Network of HRD) program (IITP-2024-RS-2022–00156439) supervised by the IITP (Institute of Information & Communications Technology Planning & Evaluation).”

The original Article has been corrected.

