# Supplementary material for: Cardiovascular outcomes in Parkinson’s disease patients from a retrospective cohort study
Source: Sci Rep. 2024 Sep 20;14:21928. doi: 10.1038/s41598-024-72549-y (PMC11415384; doi:10.1038/s41598-024-72549-y)
Supplement: Supplementary file 1 — Supplementary Tables. [file 41598_2024_72549_MOESM1_ESM.docx]

**SUPPLEMENTAL TABLE S1.** Baseline characteristics of PD patients after PS matching

|  | **PD**  **(n = 1,194)** | **Non-PD**  **(n = 4,574)** | **p-value** | **SMD** |
| --- | --- | --- | --- | --- |
| Age (years) | 73.1 ± 9.9 | 72.5 ± 10.2 | 0.09 | 0.05 |
| Male (n, %) | 527 (44.1) | 2,083 (45.5) | 0.39 | 0.03 |
| Alcohol (n, %) | 215 (18.0) | 761 (16.6) | 0.26 | 0.04 |
| Smoking (n, %) | 129 (10.8) | 493 (10.8) | 0.98 | <0.01 |
| Hypertension (n, %) | 777 (65.1) | 3,037 (66.4) | 0.39 | -0.03 |
| Diabetes (n, %) | 516 (43.2) | 2,008 (43.9) | 0.67 | -0.01 |
| Dyslipidemia (n, %) | 760 (63.7) | 2,966 (64.8) | 0.44 | -0.02 |
| CKD (n, %) | 295 (24.7) | 1,153 (25.2) | 0.72 | -0.01 |
| Atrial fibrillation (n, %) | 75 (6.3) | 276 (6.0) | 0.75 | 0.01 |
| Prior myocardial infarction (n, %) | 10 (0.8) | 41 (0.9) | 0.85 | <0.01 |
| Prior heart failure (n, %) | 40 (3.4) | 171 (3.7) | 0.52 | -0.02 |
| Prior stroke (n, %) | 233 (19.5) | 867 (19.0) | 0.66 | 0.01 |
| Prior PCI (n, %) | 30 (2.5) | 124 (2.7) | 0.7 | -0.01 |
| Systolic blood pressure (mmHg) | 127.8 ± 17.6 | 128.1 ± 16.5 | 0.57 | -0.02 |
| Diastolic blood pressure (mmHg) | 74.9 ± 11.3 | 73.7 ± 11.6 | <.01 | 0.11 |
| Pulse rate (bpm) | 79.6 ± 13.3 | 80.1 ± 21.1 | 0.32 | -0.03 |
| Body mass index | 25.0 ± 18.9 | 25.1 ± 17.3 | 0.89 | <0.01 |
| Hoehn-Yahr stage | 2.4 ± 0.9 | - | - | - |
| UPDRS scale III | 27.2 ± 14.4 | - | - | - |
| **Anti-Parkinson drugs** | | | | |
| Levodopa/COMT inhibitor (n, %) | 1,068 (89.5) | - | - | - |
| Dopamine agonist (n, %) | 544 (45.6) | - | - | - |
| MAO-B inhibitor (n, %) | 487 (40.8) | - | - | - |
| Amantadine (n, %) | 223 (18.7) | - | - | - |
| Anticholinergics (n, %) | 333 (27.9) | - | - | - |
| **Anti-hypertensive medication** | | | | |
| RAS inhibitor (n, %) | 490 (41.0) | 1,932 (42.2) | 0.45 | -0.02 |
| DHP-CCB (n, %) | 432 (36.2) | 1,701 (37.2) | 0.52 | -0.02 |
| Beta-blocker (n, %) | 449 (37.6) | 1,722 (37.7) | 0.98 | <0.01 |
| Diuretics (n, %) | 298 (25.0) | 1,133 (24.8) | 0.89 | <0.01 |
| **Anti-lipidemic medication** | | | | |
| Statin (n, %) | 527 (44.1) | 2,048 (44.8) | 0.69 | -0.01 |
| **Anti-diabetic medication** | | | | |
| OHA (n, %) | 251 (21.0) | 1,044 (22.8) | 0.18 | -0.04 |
| Insulin (n, %) | 125 (10.5) | 513 (11.2) | 0.46 | -0.02 |
| **Laboratory finding** | | | | |
| Potassium (mEq/L) | 4.2 ± 0.4 | 4.2 ± 0.4 | 0.92 | <0.01 |
| Hemoglobin (g/dL) | 13.1 ± 1.6 | 13.1 ± 1.7 | 0.49 | -0.02 |
| Creatinine (mg/dL) | 0.9 ± 0.3 | 0.9 ± 0.5 | 0.71 | -0.01 |
| eGFR (mL/min/1.73m2) | 80.4 ± 22.7 | 81.0 ± 21.8 | 0.40 | -0.03 |
| Total cholesterol (mg/dL) | 166.9 ± 38.1 | 166.6 ± 41.0 | 0.85 | <0.01 |
| HDL-cholesterol (mg/dL) | 49.0 ± 12.8 | 49.1 ± 12.3 | 0.73 | -0.01 |
| LDL-cholesterol (mg/dL) | 102.6 ± 32.3 | 103.7 ± 34.1 | 0.32 | -0.03 |
| Triglyceride (mg/dL) | 120.1 ± 64.0 | 121.0 ± 60.7 | 0.68 | -0.01 |
| hsCRP (mg/L) | 1.0 ± 1.8 | 1.5 ± 2.1 | <.01 | -0.29 |
| Glucose (mg/dL) | 121.8 ± 41.4 | 121.8 ± 47.7 | 0.97 | <0.01 |
| HbA1c (%) | 6.1 ± 1.1 | 6.4 ± 1.3 | <.01 | -0.18 |
| SCORE2 (n, %)     low-moderate risk     high risk     very high risk | 183 (15.3)  308 (25.8)  703 (58.9) | 764 (16.7)  1,199 (26.2)  2,611 (57.1) | 0.43 | 0.06 |
| CV risk group* (n, %)     low-moderate risk     high risk     very high risk | 185 (15.5)  436 (36.5)  573 (48.0) | 699 (15.3)  1,593 (34.8)  2,282 (49.9) | 0.48 | 0.04 |

Values are presented as mean ± standard deviation or number (%).

CKD, chronic kidney disease; COMT, Catechol-O-methyltransferase; CV, cardiovascular; DHP-CCB, dihydropyridine calcium channel blocker; eGFR, estimated glomerular filtration rate; HbA1c, haemoglobin A1c; HDL, high-density lipoprotein; hsCRP, high-sensitivity C-reactive protein; LDL, low-density lipoprotein; MAO-B, monoamine oxidase B; PCI, percutaneous coronary intervention; PD, Parkinson’s disease; RAS, renin-angiotensin system; OHA, oral hypoglycemic agents; SCORE2, systematic coronary risk assessment 2; SMD, standardised mean difference; UPDRS, Unified Parkinson’s Disease Rating Scale.

**SUPPLEMENTAL TABLE S2.** Cardiovascular risk groups

| Very high risk | 1. Prior MI or stroke or prior PCI 2. DM & CKD  - DM & eGFR <45 - DM & eGFR 45-59 & ACR 30-300 - DM & ACR >300 - DM & eGFR 45-59 & (U/A protein +/- or 1+) - DM & (U/A protein 2+ or more)  1. CKD  - eGFR <30 - eGFR 30-44 & ACR >30 - eGFR 30-44 & (U/A protein +/- or more)  1. Old age  - Age ≥70, SCORE2 ≥15% - Age 50-69, SCORE2 ≥10% - Age <50, SCORE2 ≥7.5% |
| --- | --- |
| High risk | 1. DM 2. CKD  - eGFR 30-44 - eGFR 45-59 & ACR 30-300 - eGFR ≥60 & ACR >300 - eGFR 45-59 & (U/A protein +/- or 1+) - eGFR ≥60 & (U/A protein 2+ or more)  1. Old age  - Age ≥ 70, SCORE2 7.5~14.99% - Age 50-69, SCORE2 5~9.99% - Age <50, SCORE2 2.5~7.49% |
| Low-moderate risk | All other patients. |

ACR, urine albumin-to-creatinine ratio; CKD, chronic kidney disease; DM, diabetes mellitus; eGFR, estimated glomerular filtration rate (mL/min/1.73m^2^); HF, heart failure; MI, myocardial infarction; PCI, percutaneous coronary intervention; SCORE2, systematic coronary risk assessment 2; U/A, urine analysis.

**SUPPLEMENTAL TABLE S3**. ICD-10 codes used for clinical diagnoses

| **Clinical diagnosis** | **ICD-10 codes** |
| --- | --- |
| Parkinson’s disease | G20, G21.1, G23.2, G23.3, G90.3 |
| Other Parkinsonism | G21.1 |
| Multiple system atrophy | G23.2, G23.3, G90.3 |
| Progressive supranuclear ophthalmoplegia | G23.1 |
| Lung cancer | C34 |
| Gastric cancer | C16 |
| Liver cancer | C22 |
| Colorectal cancer | C18, C19, C20 |
| Prostate cancer | C61 |
| Breast cancer | C50 |
| Pneumonia | J09, J10, J11, J12, J13, J14, J15, J16, J17, J18 |
| Urinary tract infection/cystitis | N30, N39.0 |

ICD, international Classification of Diseases.

**SUPPLEMENTAL TABLE S4.** Cardiovascular outcomes according to PD status before propensity score matching

|  | **PD**  **(n = 1,211)** | **Non-PD**  **(n = 86,715)** | ***p-value** |
| --- | --- | --- | --- |
| MACE | 207 (17.1%) | 11113 (12.8%) | <0.01 |
| CV death | 14 (1.2%) | 412 (0.5%) | <0.01 |
| Stroke | 16 (1.3%) | 563 (0.7%) | 0.02 |
| Myocardial infarction | 23 (1.9%) | 2654 (3.1%) | <0.01 |
| Hospitalization for HF | 197 (16.3%) | 9090 (10.5%) | <0.01 |
| All-cause death | 35 (2.9%) | 1452 (1.7%) | 0.01 |

Values are presented as number (%).

*p-value for the log-rank test.

MACE is a composite of CV death, stroke, myocardial infarction and hospitalization for heart failure.

CV, cardiovascular; HF, heart failure; MACE, major adverse cardiovascular events; PD, Parkinson’s disease.

**SUPPLEMENTAL TABLE S5a.** Multivariable analysis for MACE among PD patients using the H&Y scale for PD severity

|  | **HR (95% CI)** | **P-value** |
| --- | --- | --- |
| Middle tertile for H&Y scale | 0.88 (0.52-1.47) | 0.624 |
| Highest tertile for H&Y scale | 1.19 (0.67-2.10) | 0.561 |
| Age | 1.02 (1.00-1.05) | 0.034 |
| Sex | 1.11 (0.76-1.61) | 0.592 |
| Smoking status | 1.57 (0.92-2.69) | 0.101 |
| Drinking status | 1.42 (0.88-2.30) | 0.153 |
| Hypertension | 0.89 (0.54-1.47) | 0.649 |
| DM | 1.18 (0.85-1.65) | 0.314 |
| CKD | 1.84 (1.30-2.60) | <0.001 |
| Dyslipidaemia | 1.05 (0.67-1.63) | 0.838 |
| AF | 1.66 (0.97-2.82) | 0.063 |
| Prior HF | 9.53 (2.37-38.35) | 0.001 |
| Prior MI | 1.95 (1.00-3.80) | 0.051 |
| Prior Stroke | 1.08 (0.74-1.58) | 0.697 |
| Statin | 0.83 (0.54-1.27) | 0.388 |
| Diuretics | 1.47 (0.98-2.20) | 0.064 |
| RAS | 1.13 (0.70-1.83) | 0.606 |
| DHP-CCB | 1.37 (0.89-2.10) | 0.150 |
| Beta-blocker | 1.29 (0.91-1.81) | 0.151 |

Analysis by Cox proportional hazards model.

AF, atrial fibrillation; CI, confidence interval; CKD, chronic kidney disease; DHP-CCB, dihydropyridine calcium channel blockers; DM, diabetes mellitus; HF, heart failure; HR, hazard ratio; H&Y, Hoehn and Yahr; MACE, major adverse cardiovascular events; MI, myocardial infarction; PD, Parkinson’s disease; PS, propensity score; RAS, renin-angiotensin system.

**SUPPLEMENTAL TABLE S5b.** Multivariable analysis for MACE among PD patients using the UPDRS-III scale for PD severity

|  | **HR (95% CI)** | **P-value** |
| --- | --- | --- |
| Middle tertile for UPPRS-III score | 0.98 (0.61-1.55) | 0.9185 |
| Highest tertile for UPDRS-III score | 1.08 (0.65-1.81) | 0.7696 |
| Age | 1.03 (1.01-1.05) | 0.0175 |
| Sex | 1.14 (0.78-1.67) | 0.4881 |
| Smoking status | 1.64 (0.95-2.83) | 0.0783 |
| Drinking status | 1.45 (0.88-2.38) | 0.1414 |
| Hypertension | 0.87 (0.52-1.44) | 0.5819 |
| DM | 1.20 (0.85-1.68) | 0.2951 |
| CKD | 1.83 (1.29-2.60) | 0.0007 |
| Dyslipidaemia | 1.08 (0.69-1.68) | 0.7388 |
| AF | 1.69 (1.00-2.85) | 0.0504 |
| Prior HF | 10.4 (2.68-40.44) | 0.0007 |
| Prior MI | 2.08 (1.09-3.98) | 0.027 |
| Prior Stroke | 1.05 (0.71-1.56) | 0.7958 |
| Statin | 0.79 (0.51-1.22) | 0.2848 |
| Diuretics | 1.47 (0.98-2.21) | 0.0651 |
| RAS | 1.15 (0.71-1.87) | 0.5765 |
| DHP-CCB | 1.39 (0.90-2.15) | 0.137 |
| Beta-blocker | 1.29 (0.91-1.82) | 0.1561 |

Analysis by Cox proportional hazards model.

AF, atrial fibrillation; CI, confidence interval; CKD, chronic kidney disease; DHP-CCB, dihydropyridine calcium channel blockers; DM, diabetes mellitus; HF, heart failure; HR, hazard ratio; MACE, major adverse cardiovascular events; MI, myocardial infarction; PD, Parkinson’s disease; PS, propensity score; RAS, renin-angiotensin system; UPDRS-III, Unified Parkinson’s Disease Rating Scale.

**SUPPLEMENTAL TABLE S6.** Non-cardiovascular outcomes according to PD status after propensity score matching

|  | **PD**  **(n =1,194)** | **Non-PD**  **(n = 4,574)** | ***p-value** |
| --- | --- | --- | --- |
| **Malignant neoplasms** | | | |
| Lung cancer | 8 (0.7) | 77 (1.7) | <0.01 |
| Gastric cancer | 15 (1.3) | 139 (3.0) | <0.01 |
| Liver cancer | 9 (0.8) | 138 (3.0) | <0.01 |
| Colorectal cancer | 9 (0.8) | 131 (2.9) | <0.01 |
| Prostate cancer | 20 (1.7) | 93 (2.0) | 0.40 |
| Breast cancer | 13 (1.1) | 81 (1.8) | 0.09 |
| **Infections** | | | |
| Pneumonia | 60 (5.0) | 174 (3.8) | 0.10 |
| Urinary tract infection/cystitis | 86 (7.2) | 187 (4.1) | <0.01 |
| Admission due to pneumonia | 32 (2.7) | 71 (1.6) | 0.01 |
| Admission due to urinary tract infection/cystitis | 28 (2.4) | 27 (0.6) | <0.01 |
| 30-day mortality from pneumonia | 7 (0.6) | 18 (0.4) | 0.38 |
| 30-day mortality from urinary tract infection/cystitis | 2 (0.2) | 2 (0.04) | 0.15 |

Values are presented as number (%).

*p-value for the log-rank test.

PD, Parkinson’s disease.

**SUPPLEMENTAL TABLE S7**. Effect of PD on cardiovascular and non-cardiovascular outcomes in the PS-matched cohort

|  | **HR (95% CI)** | **P-value** |
| --- | --- | --- |
| **CV outcomes** |  |  |
| MACE | 1.01 [0.86;1.17] | 0.93 |
| CV death | 1.26 [0.72;2.23] | 0.416 |
| Stroke | 1.17 [0.67;2.05] | 0.586 |
| MI | 0.88 [0.56;1.39] | 0.593 |
| HF hospitalization | 1.05 [0.90;1.24] | 0.51 |
| All-cause death | 0.85 [0.59;1.23] | 0.399 |
| **Infections** |  |  |
| Pneumonia | 1.27 [0.94;1.71] | 0.12 |
| UTI | 1.73 [1.33;2.24] | <0.001 |
| Pneumonia 30-day mortality | 1.48 [0.62;3.53] | 0.383 |
| UTI 30-day mortality | 3.79 [0.53;26.93] | 0.182 |
| **Malignant neoplasms** |  |  |
| Lung cancer | 0.40 [0.19;0.83] | 0.0136 |
| Gastric cancer | 0.39 [0.22;0.67] | <0.001 |
| Colorectal cancer | 0.26 [0.13;0.51] | <0.001 |
| Liver cancer | 0.25 [0.13;0.49] | <0.001 |
| Prostate cancer | 0.83 [0.51;1.34] | 0.44 |
| Breast cancer | 0.57 [0.31;1.04] | 0.0685 |

CI, confidence interval; CV, cardiovascular; HF, heart failure; HR, hazard ratio; MACE, major adverse cardiovascular events; MI, myocardial infarction; PD, Parkinson’s disease; PS, propensity score; UTI, urinary tract infection.
